# Determination of Anti-drug Antibody Affinity in Clinical Study Samples Provides a Tool for Evaluation of Immune Response Maturation

**DOI:** 10.1208/s12248-022-00759-1

**Published:** 2022-11-02

**Authors:** Alison Joyce, Christopher Shea, Zhiping You, Boris Gorovits, Christopher Lepsy

**Affiliations:** 1 Pfizer, Inc., Worldwide Research & Development, Biomedicine Design, 1 Burtt Road, Andover, Massachusetts USA; 2 Pfizer, Inc, Worldwide Research & Development, Early Clinical Development, 1 Burtt Road, Andover, Massachusetts USA; 3 Present Address: Development Sciences, Sana Biotechnology, Inc., 300 Technology Square, Cambridge, Massachusetts USA

**Keywords:** Immunogenicity, Biotherapeutics, Anti-drug antibody, Affinity maturation, Affinity ligand binding assay

## Abstract

Characterization of clinical anti-drug antibody (ADA) responses to biotherapeutics can be important to understanding the consequences of immunogenicity. ADA are expected to be polyclonal, with composition and affinities that evolve over time. Measuring ADA binding affinity can be complicated by the polyclonal nature of response, residual drug in sample, and low ADA levels. We developed a novel workflow to determine the apparent ADA affinity (*K*_*D*_) against a monoclonal antibody biotherapeutic, PF-06480605. An affinity capture elution pre-treatment step was used to isolate ADA and remove residual drug interference from samples. Solution-phase equilibrium incubation was performed using drug and sample ADA as variable and fixed binding interactants, respectively. Unbound ADA concentration was measured using a Singulex Erenna ligand-binding assay (LBA) method. Apparent ADA *K*_*D*_ values were calculated using a custom R Shiny algorithm. *K*_*D*_ values determined for ADA positive samples showed good correlation with other immunogenicity parameters, including titers and neutralizing antibody (NAb) activity with a general increase in affinity over time, indicative of a maturing immune response. Time of onset of high affinity responses (*K*_*D*_ < 100 pM) varied between patients, ranging from 16 to 24 weeks. Antibody responses appeared monophasic at earlier time points, trending towards a biphasic response with a variable transition time and general increase in proportion of high affinity ADA over time. Herein, we provide a novel, sensitive bioanalytical method to determine the *K*_*D*_ of ADA in clinical samples. The observed decrease in ADA *K*_*D*_ is consistent with evidence of a maturing immune response.

**Graphical Abstract:**

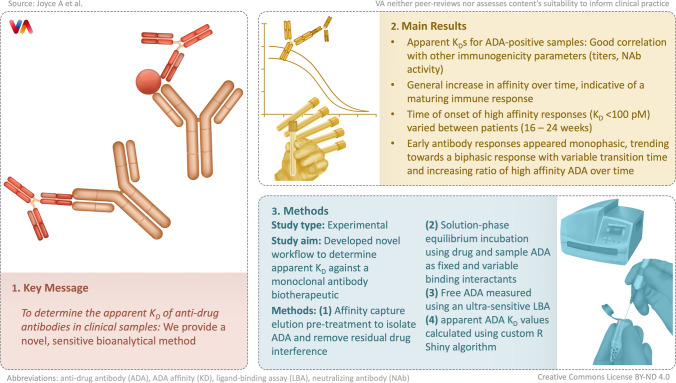

## Introduction

Immunogenicity against biotherapeutics can impact their safety and efficacy (1–4). Current approaches to characterize the clinical immune response to biotherapeutics include assessments of onset, duration, and magnitude of ADA response (titer) using qualitative or semi-quantitative methods, typically utilizing a LBA format. Regulatory agencies also recommend assessment of additional characteristics, including neutralizing antibody (NAb) activity, specificity of ADA response and isotyping when warranted (5–7).

The affinity dissociation constant (*K*_*D*_) of ADA is an important attribute which can represent the kinetics of antibody development and has rarely been included in immunogenicity assessments of biotherapeutics. European Medicines Agency (EMA) guidance states affinity characterization of ADA response may be required on a case-by-case basis, and assays used for these measurements should be qualified for their intended purpose (8). In the case of FVIII inhibitors, longitudinal analysis of relative affinity of clinical ADA has been assessed using ELISA and correlated to ADA titer and neutralizing activity (9). To date, there have been no published data on the evaluation of relative affinity of clinical ADA against a monoclonal antibody biotherapeutic over time, correlation with ADA titer and NAb incidence data and impact to the PK, and efficacy and safety of the drug.

PF-06480605 is an anti-tumor necrosis factor-like ligand 1A (TL1A) human IgG1 antibody with the potential to treat moderate to severe ulcerative colitis (UC) and Crohn’s Disease. TUSCANY is a phase 2a study which evaluated safety, tolerability, efficacy, PK, and immunogenicity of PF-06480605 in patients with moderate to severe UC. Immunogenicity assessments included ADA and NAb determination using LBA and cell-based immunogenicity assays, respectively. Despite an 82% ADA and 10% NAb positivity rate, no statistically significant effects of ADA status on endoscopic improvement or other various clinical endpoints were found. The PK profile also appeared unaffected. However, a trend towards reduction in total soluble TL1A levels was observed in ADA and NAb positive patients compared to ADA and NAb negative subjects (10). To further characterize the immune response in selected ADA positive patients, the apparent *K*_*D*_ of ADA for drug across the time course of study was conducted.

ADA and NAb assay signals are dependent on both affinity and concentration of the ADA in the sample. Challenges to affinity determination in clinical ADA samples include a combination of assay sensitivity due to the relatively low concentrations of ADA, matrix interference from serum proteins, residual drug, and the polyclonal nature of the ADA response. There are many technologies available to determine the *K*_*D*_ between two binding interactants, and these are reviewed in detail elsewhere (11–14). Methods include surface-based methodologies, such as Biacore™ and Octet that use the kinetic properties *k*_*on*_ and *k*_*off*_ to calculate equilibrium constants *K*_*D*_ and *K*_*A*_ (ratio of *k*_*off*_/*k*_*on*_ = *K*_*D*_ = 1/*K*_*A*_, where *K*_*D*_ and *K*_*A*_ are the equilibrium dissociation and association constants, respectively). However, sensitivity constraints as well as biological matrix and surface effects may preclude application of these methods for evaluation of ADA affinity in study incurred clinical samples. An alternative approach utilizes concentration determination of two binding interactants at equilibrium in solution phase. Free (unbound) concentration of fixed interactant is measured using a LBA and plotted against known concentrations of the variable interactant, allowing determination of *K*_*D*_ (11, 15) (12, 14). Technologies such as KinExA® and Gryolab® use this solution-based approach for *K*_*D*_ determination and can measure significantly lower *K*_*D*_ values compared to surface-based methodologies. In addition, KinExA and Gyrolab offer built-in software capabilities for calculating *K*_*D*_ values. KinExA, however, is generally a low-throughput instrument and needs a relatively large sample volume, while Gyrolab utilizes small sample volume and a comparatively quick assay run time. Both KinExA and Gyrolab can measure a wide range of *K*_*D*_ values, from low pM or fM to mM. However, when attempting to detect an apparent *K*_*D*_ of ADA in clinical study samples, where ADA concentrations may be low, the sensitivity of the platform becomes increasingly important. Initial attempts to measure apparent *K*_*D*_ of clinical sample ADA using this approach on Gyrolab proved unsuccessful due to sensitivity constraints. McDonald et al. used an ultrasensitive microparticle LBA with single molecule fluorescence imaging to assess the apparent affinity of anti-SARS-CoV-2 antibodies in COVID-19 patient plasma (16). Singulex Erenna® uses a single molecule counting technology to detect minute levels of protein based on a specific LBA and offers a useful alternative for detecting low amounts of ADA in clinical samples (17), (18).

In this study, clinical ADA samples over a time course from 15–183 days were obtained from a subset of patients treated with PF-06480605. ADA content was first enriched using an affinity-capture-elution (ACE) procedure optimized for improved analyte recovery and assay drug tolerance using a mouse anti-idiotype monoclonal antibody against PF-06480605 positive control (PC) reagent. Pre-treated enriched PC and ADA samples collected from patients treated with PF-06480605 were incubated with varying amounts of unlabeled PF-06480605 until equilibrium was reached. Free (unbound) ADA concentration was then measured using a specific Singulex Erenna bridging ADA assay. Data was analyzed to determine apparent *K*_*D*_ values using a custom-built R Shiny application. Apparent *K*_*D*_ values, monophasic vs biphasic profiles, and the proportion of low affinity vs. high affinity ADA populations within each sample were compared across the time course for each available patient. In addition, ADA titers, NAb status, and apparent ADA concentration were also correlated to apparent *K*_*D*_.

## Materials and Methods

### Reagents (Singulex Erenna Assay)

PF-06480605 (unlabeled, biotinylated, and Alexa Fluor 647–labeled), drug-coated paramagnetic beads, and mouse anti-idiotype monoclonal antibody against PF-06480605 as PC, were all developed by Pfizer (Andover, MA). The following buffers were all supplied by Pfizer: storage buffer (phosphate-buffered saline, calcium, and magnesium–free (PBS-CMF) with 0.05% Tween®20 and 1% bovine serum albumin, pH 7.2 (PBST/1% BSA), Tris High Salt Wash Buffer (THST) (50 mM Tris, 1 mM glycine, 500 mM NaCl, 0.05% Tween®20), acid dissociation buffer (100 mM glycine, pH 2.0), and neutralization buffer (1 M Tris, pH 9.0). Pooled normal human serum was obtained from BioIVT (Westbury, NY). SMC™ Capture Antibody Labeling Kit (cat #03–0077-02) and SMC™ Bead–Based Assay Development Kit (cat #03–0178-00) were purchased from Millipore Sigma (Burlington, MA). A total of 384-well polypropylene microtiter plates (cat #264573) and Axygen™ polypropylene V-bottom 96-well microplates (cat #P-96-450 V-C) were both purchased from Thermo Fisher Scientific (Waltham, MA). The instrument used for ligand-binding affinity assay analysis in patient samples was the Erenna® Immunoassay System using SMC™ technology from Millipore Sigma (Burlington, MA).

### Reagents (Gyrolab Assay)

PF-06480605 (unlabeled, biotinylated, and Alexa Fluor 647–labeled), drug-coated paramagnetic beads, and mouse anti-idiotype monoclonal antibody against PF-06480605 as PC, were all developed by Pfizer (Andover, MA). The following buffers were all supplied by Pfizer: storage buffer (phosphate-buffered saline, calcium, and magnesium–free (PBS) with 0.05% Tween®20 and 1% bovine serum albumin, pH 7.2 (PBST/1% BSA). Tris High Salt Wash Buffer (THST) (50 mM Tris, 1 mM glycine, 500 mM NaCl, 0.05% Tween®20), acid dissociation buffer (100 mM glycine, pH 2.0), and neutralization buffer (1 M Tris, pH 9.0). Pooled normal human serum was obtained from BioIVT (Westbury, NY). Wash station 1 buffer (PBS + 0.01% Tween®20) was prepared by Pfizer. Wash station 2 buffer (Gyrolab wash buffer pH 11, cat #P0020096), Gyrolab Bioaffy 1000 nanoliter CDs (cat #P0004253), Rexxip F buffer (cat #P0004825), 96-well PCR plates (cat #P004861), and microplate foil seals (cat #P003313) were all purchased from Gyros Protein Technologies (Warren, NJ). The instrument used for ADA assay development was the Gyrolab xPand with Gyrolab affinity software module (cat# P0020413).

### Clinical Samples for Analysis

Samples were obtained from a subset of patients participating in the TUSCANY trial, a phase 2a study which evaluated safety, tolerability, efficacy, PK, and immunogenicity of PF-06480605 in patients with moderate to severe UC (10). Samples selected for affinity analysis were limited, based on availability of sufficient sample volumes and demonstration of PF-06480605 ADA log(10) titer ≥ 2.0. Using this criterion, a total of 7 patients had evaluable samples. Samples generally spanned multiple time points ranging from 15 to 183 days after drug administration, thereby enabling characterization of *K*_*D*_ over time. Three patients had available samples spanning 5–7 time points within this timeframe, and one patient had available samples from 3 time points over a more limited timeframe (141–183 days). Three additional patient samples from 1 or 2 time points each were included in measurements.

### Bead Labeling with PF-06480605 for ACE Procedure and ADA LBA Capture Reagent

PF-06480605-coupled beads for the ACE procedure were produced at Pfizer (Andover, MA). Briefly, sulfo N-hydroxysuccinimide (NHS) activated carboxyl-coated paramagnetic beads (2 × 10^9^ beads/mL) were directly conjugated with 0.45 mg PF-06480605 using EDC (1-ethyl-3-(3-dimethylaminopropyl)carbodiimide hydrochloride) chemistry through primary amines. For the Singulex Erenna ADA LBA, biotinylated PF-06480605 was coated on streptavidin paramagnetic beads using the SMC™ Capture Antibody Labeling Kit at 6.25 µg IgG/mg beads. The bead solution was incubated on an inversion rotator for 60 min at room temperature. Coated beads were washed 5 times, and beads stored in kit coating bead buffer at 2–8°C until use.

### Assay Procedure

**Fig. 1:**
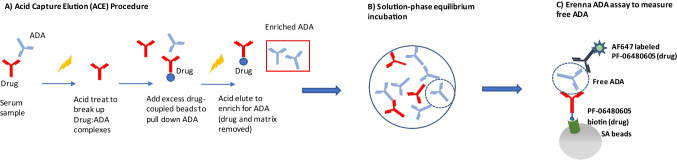
ADA affinity assay workflow. (**A**) An affinity capture elution (ACE) method was used to enrich ADA from serum matrix and improve drug tolerance. (**B**) Enriched ADA at one dilution or PC was incubated with a range of PF-06480605 concentrations to reach equilibrium. (**C**) Solution-phase equilibrium samples were assayed using a bridging ADA LBA to assess free ADA levels against PF-06480605. Data were subsequently analyzed using a customized R Shiny application for apparent *K*_*D*_ determination

The ADA affinity assay procedure is comprised of 3 steps as listed below and depicted in Fig. 1. Steps 1 and 2 are common to both Singulex Erenna and Gyrolab ADA assays (sample pre-treatment steps). Step 3 has LBA platform-specific assay procedure descriptions. Both platforms use the same biotinylated and Alexa Fluor 647–labeled PF-06480605 as capture and detection reagents, respectively, and the same MAb PC.

#### Step 1: ACE Procedure for ADA and PC Enrichment

The PC was prepared by spiking an idiotype specific anti-PF-06480605 mouse MAb into neat (100%) pooled normal human serum for a final concentration of 160 µg/mL and incubating for 30 min at room temperature (RT) on a shaker. The PC preparation was tested with and without PF-06480605 (25 µg/mL). The ACE procedure was performed on the PC samples and clinical study samples to enrich ADA concentrations and improve drug tolerance by reducing residual PF-06480605. One milliliter of PF-06480605 cross-linked paramagnetic beads was placed on a magnetic stand and the storage buffer was removed. Beads were acid washed to reduce drug leaching during the ACE procedure. Beads were resuspended in 500 µL of 100 mM glycine pH 2.0 and rotated end-over-end for 30 min at RT. Following incubation, the beads were placed on a magnetic stand and glycine buffer was removed, followed by resuspension in PBST/1% BSA and letting stand for 1 min. This wash procedure was repeated 2 more times and beads were stored in PBST/1% BSA at 2–8°C until use. To ACE-treat the study samples and PC preparation, 50 µL of sample was added to 25 µL Assay Buffer from the SMC™ Bead–Based Assay Development Kit in a polypropylene V-bottom plate. Fifty microliters of 100 mM glycine pH 2.0 was subsequently added and the solution in the plate was incubated for 30 min at RT on shaker. Following the incubation, 60 µL of the drug-coated beads was added to the plate wells containing the treated study samples and PC preparation, followed by addition of 14 µL of 1 M Tris pH 9.0 to neutralize the mixture. The plate was then sealed and placed on a shaker overnight at RT. After the incubation, the plate was placed on a BioTek ELx405™ plate washer with magnetic plate adapter and washed 6 times with 300 µL 1X THST buffer. Elution of the affinity captured ADA or PC was performed by adding 50 µL of 100 mM glycine pH 2.0 to the pelleted beads in each well followed by a 10-min incubation at RT on a plate shaker. The plate was placed on a magnetic adapter and beads were pelleted for 1 min. Fifty microliters of eluted material was removed and neutralized with 7 µL of 1 M Tris pH 9.0 in separate tubes. Storage buffer (PBST/1% BSA) was added in the amount of 25 µL and tubes were stored frozen at −70°C until use.

#### Step 2: Preparation of ADA-Enriched Solution-Based Drug Equilibrium Samples for Affinity Determination

A solution-based equilibrium approach was used to measure the apparent affinity of ADA binding to drug. Briefly, enriched ADA from patient samples or PC-spiked serum was kept at a constant dilution (fixed interactant) and incubated with a broad concentration range of unlabeled PF-06480605 (variable interactant) until equilibrium was reached. Equilibrium time was determined to be approximately 24 h, based on the MAb PC (data not shown). Initially, the drug stock was diluted serially 5-fold for a total of eight dilutions ranging from 1000 to 0.01 ng/mL using Standard Diluent from the SMC™ Bead–Based Assay Development Kit and served as the variable affinity interactant. The ACE-processed samples and PC were fixed to one dilution as follows: processed samples were diluted 20-fold and processed PC diluted to 1:15,000 in Standard Diluent. Affinity solution samples were prepared by mixing 50 µL of each of the dilutions of the PF-06480605 to 50 µL of the ACE-processed patient samples or PC and incubated on a plate shaker at RT until equilibrium was reached (at least 24 h).

#### Step 3(A): ADA Sample Analysis on the Singulex Erenna Free ADA Ligand-Binding Assay

The equilibrated affinity solution sample set was analyzed with a bead-based free ADA bridging LBA using Singulex Erenna. The bridging ADA format used PF-06480605 to capture and Alexa fluorophore labeled PF-06480605 to detect and measure free (unbound) ADA in the affinity solution samples (Fig. 1C). A reference curve using the ACE-processed MAb PC was also included in each run to evaluate apparent ADA concentration in the processed affinity samples. The concentration of the processed MAb PC was based on an approximated 30% recovery post-ACE procedure, which was determined during ACE method optimization (data not shown). The % recovery of the MAb PC post-ACE procedure was determined by interpolating the ACE-treated MAb PC concentration from a MAb PC reference curve (not ACE processed) of known concentration. The apparent (estimated) ADA concentration of the processed affinity samples was determined using the same approach. A 1:20 dilution of the processed affinity samples was assayed and signal interpolated off the MAb PC reference curve to calculate apparent concentration. After the 24 h incubation step, 100 µL of the solution affinity samples, MAb PC, and the 1:20 dilution of ACE-enriched ADA samples were added to a polypropylene V-bottom plate and 100 µL of drug capture beads added to the samples and the plate was shaken at room temperature for 2 h. The plate was washed with 1× wash buffer from the SMC™ Bead–Based Assay Development Kit using a BioTek plate washer ELx 405 with magnetic bead plate adapter. The PF-06480605 Alexa Fluor 647 detector was centrifuged at 14,000 × g for 5 min and diluted in kit Assay Buffer to 10 ng/mL and filtered through a 0.2-µm syringe filter. After plate wash completion, 20 µL/well of the filtered detector was added to each well and incubated for 1 h at room temperature on a plate shaker. Post incubation, the plate was washed 2 times with magnetic plate adaptor using 1× wash buffer. Following the final aspiration on the plate washer, 11 µL/well of kit Elution Buffer B was added to the plate and placed on a plate shaker for 10 min. Ten microliters/well of kit Neutralization Buffer D was added to appropriate wells of a polypropylene 384-well plate. After 10 min of incubation, the Elution Buffer plate was placed on the magnetic plate adaptor for 2 min and 10 µL of eluted sample was transferred into the 384-well reading plate with neutralization buffer. The 384-well reading plate was centrifuged at 1100 × g for 5 min and heat-sealed with foil using FluidX thermosealer (Brooks Life Sciences, cat#4ti-0655). Finally, the sealed plate was read on the Erenna instrument.

**Fig. 2:**
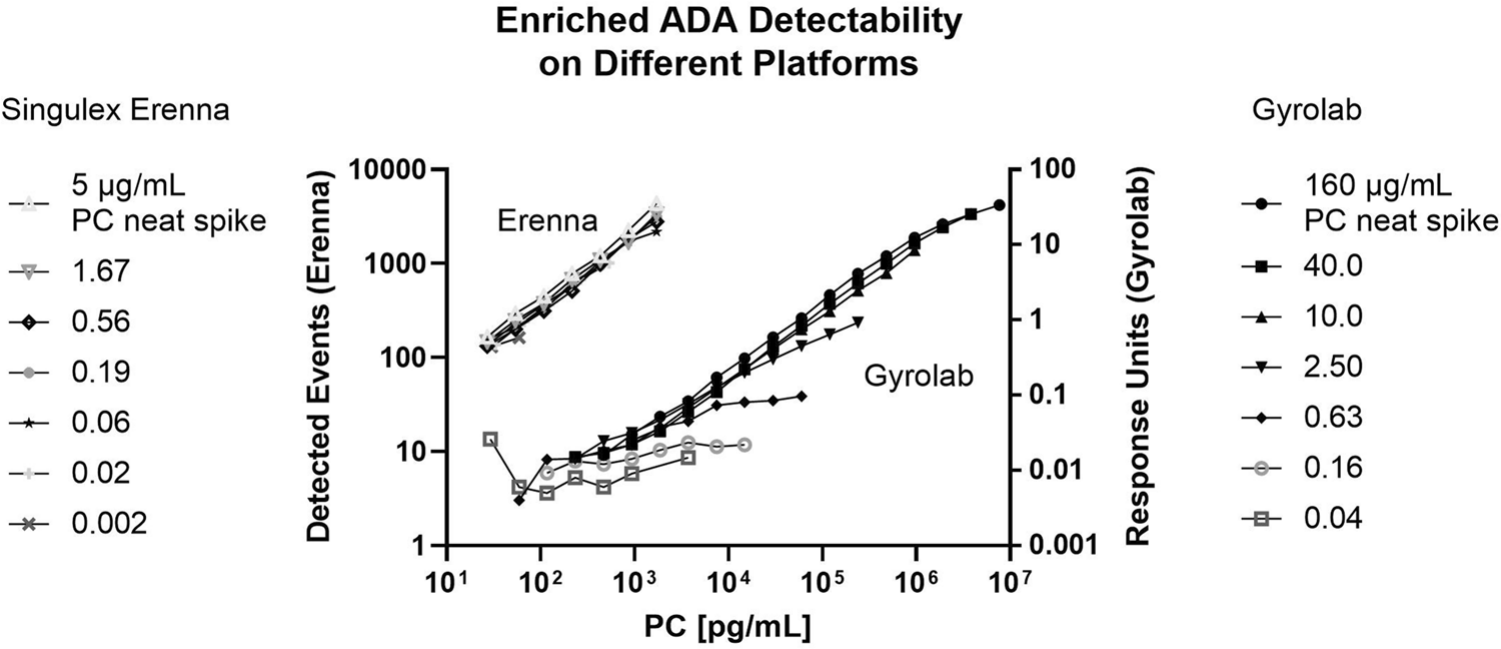
Anti-PF-06480605 PC was spiked into neat serum at a range of concentrations and processed through the ACE workflow. Resulting enriched PC preps were diluted serially and assayed on Gyrolab and Singulex Erenna using the ADA bridging assay format. Left *Y*-axis denotes Erenna assay signal as detected events, and right *Y*-axis denotes Gyrolab Response Units signal. *X*-axis denotes PC on-plate concentrations for the dilution curves in pg/mL

#### Step 3(B): ADA Analysis on the Gyrolab-Free ADA Ligand-Binding Assay

The equilibrated affinity solution sample set was analyzed with a bead-based free ADA bridging LBA using Gyrolab. The bridging ADA format used biotinylated PF-06480605 to capture and Alexa fluorophore–labeled PF-06480605 to detect and measure free (unbound) ADA in the affinity solution samples (Fig. 1C). A reference curve using the ACE-processed MAb PC was also included in each run to evaluate apparent ADA concentration in the processed affinity samples. The concentration of the processed MAb PC was estimated based on an approximated 30% recovery post-ACE procedure, which was determined during ACE method optimization (data not shown). The % recovery of the MAb PC post-ACE procedure was determined by interpolating the ACE-treated MAb PC concentration from a MAb PC reference curve (not ACE processed) of known concentration. After the 24 h incubation step, biotinylated PF-06480605 drug was diluted to 3 µg/mL in Wash Station 1 buffer, and Alexa Fluor 647–labeled PF-06480605 was centrifuged at 14,000 × g for 5 min and diluted to a final concentration of 2 µg/mL in Rexxip F buffer. Reagents and PC samples were added to the 96-well PCR plate(s) which were sealed with specific Gyrolab foil seal(s) and loaded onto the instrument with the run executed with appropriate number of Gyrolab 1000 nL Bioaffy CDs.

#### Data Analysis: Custom R Shiny Application for Calculating Apparent K_D_

Because there is no affinity module associated with the Erenna instrument, a custom application using R Shiny was developed to calculate apparent *K*_*D*_ values using the principles of binding interactants in solution at equilibrium. In addition, the polyclonal nature of the response was considered by using an approach by Stevens (19), where the response may be simplified as biclonal with the assumption that two populations (here, low and high affinity) exist in the sample, and a geometric mean of both populations could give one *K*_*D*_ readout.

The following two model equations were used for fitting data to determine apparent *K*_*D*_ and are described in detail elsewhere (19) where *Ab* is the maximum signal for the corresponding phase, *Dose* is the amount of PF-06480605 added to the equilibrium affinity solution, *K*_*A*_ is the equilibrium association constant and *K*_*D*_ is the apparent equilibrium dissociation constant:

#### Monophasic Approach

$$Response= \frac{Ab\, (1+2 \,Dose \,{K}_{A})}{{(1+Dose\, {K}_{A})}^{2}}$$

#### Biphasic Approach

$$Response= \frac{{Ab}_{1}\, (1+2\, Dose\, {K}_{A1})}{{(1+Dose {K}_{A1})}^{2}}+ \frac{{Ab}_{2}\, (1+2\, Dose\, {K}_{A2})}{{(1+Dose {K}_{A2})}^{2}}$$

Using *K*_*D*_ = 1/*K*_*A*_, we can also write the above models substituting *K*_*A*_ for *K*_*D*_ as follows:

$$Response=Ab\, (1- \frac{1}{{\left(\frac{{K}_{D}}{Dose}+1\right)}^{2}})$$

$$Response={Ab}_{1} \left(1- \frac{1}{{\left(\frac{{K}_{D1}}{Dose}+1\right)}^{2}}\right)+ {Ab}_{2}\, (1- \frac{1}{{\left(\frac{{K}_{D2}}{Dose}+1\right)}^{2}})$$

Affinity curves were fit with both monophasic and biphasic models followed by applying adjusted Akaike information criterion (AIC) to determine which model was best suited for each curve (20). The adjusted AIC considers both the number of parameters and the sample size (number of different concentrations of the variable interactant). The probability of each model is calculated to determine the best fit, with probability > 50% designated as the model fit acceptance criteria.

The monophasic model fit result is relatively straightforward and has been described in detail elsewhere (21). Briefly, the biphasic model has two separate outputs described below:

1. Proportion: The biphasic model assumed there were two affinity populations present (high and low *K*_*D*_) and had two distinct *Ab* values, one for each phase. Each phase represented a portion of the overall response. The two *Ab* values were normalized, and the output was the corresponding % proportion attributed to phase 1 (high affinity) and phase 2 (low affinity).
2. Geometric mean: The biphasic model had two *K*_*D*_*'s*, *K*_*D*_*1* and *K*_*D*_*2*, for high and low affinity populations, respectively. For simplicity, one *K*_*D*_ measurement to describe the overall affinity profile was also included. To that end, the geometric mean of *K*_*D*_*1* and *K*_*D*_*2* was computed (denoted *K*_*D(geo)*_) and used as one practical affinity measurement which accounted for both high and low affinity populations. The *K*_*D(geo)*_ fell between *K*_*D*_*1* and *K*_*D*_*2* and was generally close to the monophasic *K*_*D*_.
  The quality of the affinity curve was dependent on the free ADA LBA signal window achieved. The signal window was defined as the ratio of the lowest signal to the highest signal as the drug concentrations were varied during solution equilibrium incubations. The free ADA LBA signal window must be high enough to demonstrate a sufficient level of inhibition over the range of drug concentrations so *K*_*D*_ may be accurately calculated. The accuracy of the *K*_*D*_ value would be negatively impacted if the affinity inhibition curve was too flat. A practical acceptance test, referred to as a “flatness test,” was applied to determine if the affinity curves (both monophasic and biphasic) had an appropriate inhibition profile and signal window to generate an accurate *K*_*D*_. First, the affinity curves were fit for both monophasic and biphasic models. At every drug concentration, based on the fitted curve, there would be a predicted response value with a confidence bound limit. The upper and lower confidence bound of each prediction was computed with confidence level set at 99% across the entire drug concentration range, from the highest level to the lowest. If the lower bound of the prediction at the lowest drug concentration point (highest assay signal) overlapped with the higher bound limit at the highest drug concentration (lowest assay signal), then the responses at either end of the curve are not statistically differentiated, implying signal and/or inhibition was not obtained and the *K*_*D*_ measurement was not acceptably accurate. For the best quality *K*_*D*_ value, both monophasic and biphasic curve fits must pass the acceptance criteria.

## Results

### Analytical Platform Suitability

Two different LBA platforms (Gyrolab vs Erenna) were assessed to measure ADA *K*_*D*_ in clinical samples using a solution-based equilibrium approach. The Gyrolab was considered due to the small sample volume requirement, quick assay run time, and built-in affinity software module. The Singulex Erenna was also evaluated due to its ultra-sensitivity capabilities and concerns that enriched ADA concentrations may be too low to be detected on Gyrolab.

Experimental samples were generated either by spiking a PC into pooled human serum or from incurred study samples from 7 patients participating in the TUSCANY trial. All samples were pre-treated with an ACE procedure to enrich the ADA concentration and remove residual drug, thereby enabling affinity measurements. To prepare affinity solution samples for analysis, a dilution series of unlabeled PF-06480605 was spiked with a fixed concentration of enriched ADA from samples and incubated for 24 h. ADA not bound to drug in the affinity solution samples was detected using a bridging free ADA LBA, and the data was analyzed using a proprietary algorithm to calculate the *K*_*D*_ values.

Initially, the investigation focused on establishing a robust sample pre-treatment step that would achieve three main goals: (1) enrich the ADA concentration, (2) reduce the concentration of residual drug to reduce drug interference, and (3) reduce or remove interfering serum proteins to improve overall ADA assay performance. We applied a bead-based ACE procedure to achieve these aims and in the process assessed both the Gyrolab and Singulex Erenna analytical platforms for suitability of analysis.

Anti-PF-06480605 PC spiked into serum (+ / − drug) was used to optimize the ACE procedure, demonstrate appropriate ADA assay drug tolerance, and to determine the approximate overall sensitivity of the method. Purified PC and patient ADA samples were processed through the affinity workflow.

The ADA binding affinity evaluation was originally carried out on the Gyrolab platform because it allows for smaller sample volumes, has a fast assay time, and provides the added benefit of a built-in affinity module for *K*_*D*_ analysis. Anti-PF-06480605 PC was spiked into neat human serum at a range of concentrations (5.0–0.002 μg/mL for Erenna; 160–0.04 μg/mL for Gyrolab) and processed through the ACE workflow. Resulting enriched PC preparations were diluted and assayed on Gyrolab and Singulex Erenna using the free ADA LBA format. Enriched PC concentrations were calculated with the assumption of approximately 30% recovery after ACE procedure and accounted for dilution of the sample upon ACE treatment. Using Gyrolab, as the initial serum-spiked PC concentration decreased below the 2.5 μg/mL level, the intensity of the PC ADA signal was dramatically reduced. In contrast, when using Erenna platform, the PC ADA signal was detectable in samples with PC-spiked serum concentrations as low as 20 ng/mL (Fig. 2). The affinity assay format described herein generated reliable data on the Gyrolab for samples containing 160–2.5 μg/ml of PC material. However, we were unable to generate a suitable affinity curve using Gyrolab for several clinical samples despite high ADA titers. It was therefore determined that a higher sensitivity ADA method was required to produce a reliable and reproducible signal in the ADA assay after the ACE sample pre-treatment. Using the experimental conditions described, we demonstrated an approximate 125-fold increase in the sensitivity of ADA detection using the Singulex Erenna platform compared to Gyrolab. Consequently, the free ADA LBA assay was transferred to the Singulex Erenna platform, which has demonstrated analyte sensitivity in the sub pg/mL range, for further evaluation.

The decision to shift to the Erenna platform for enhanced sensitivity came with the need for a custom algorithm to calculate *K*_*D*_ using the free ADA LBA data, since the Erenna had no built-in module for this purpose. A custom R Shiny application was developed and PC apparent *K*_*D*_ determined using the algorithm. A comparison of the apparent *K*_*D*_ value of the PC for PF-06480605 across multiple platforms using the solution phase equilibrium approach showed the PC to have high affinity, in the low pM range, irrespective of platform used. In general, the Erenna-based *K*_*D*_ measurement of 2.0 pM for the PC was consistent with two other technologies using solution-based affinity approaches, Gyrolab and KinExA (3.0 and 15 pM *K*_*D*_, respectively). Considering platform differences including different assay formats for determining free fixed analyte concentration, these numbers were considered comparable.

**Fig. 3:**
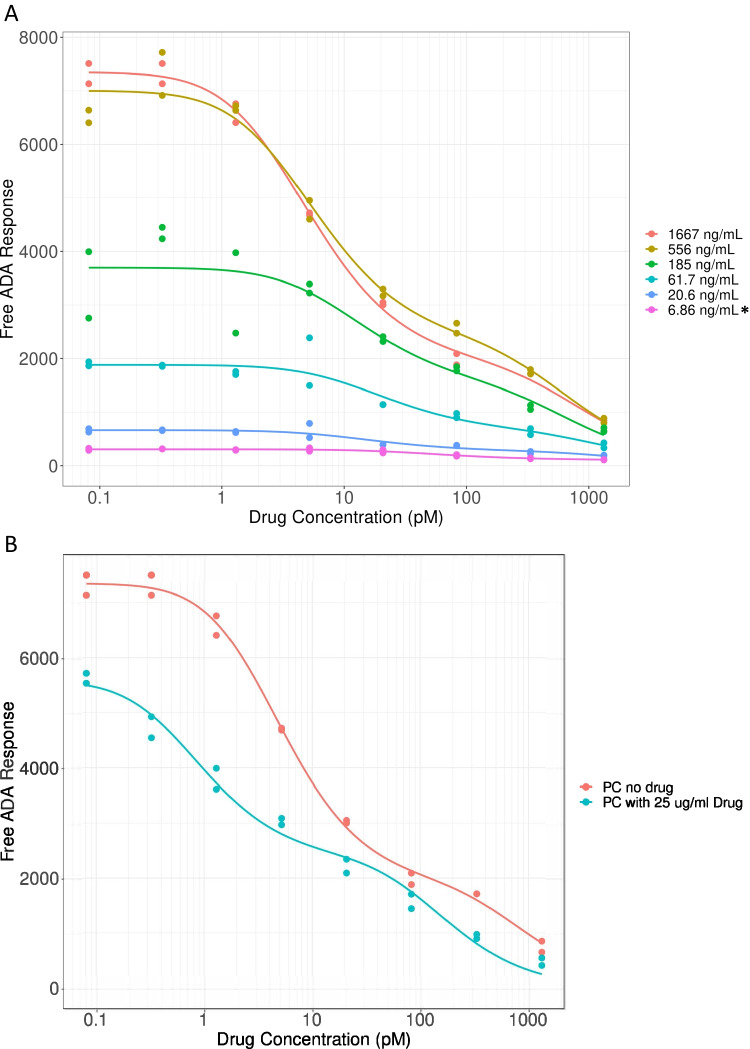
PC titration and drug tolerance testing for ADA affinity assay. (**A**) Affinity curves derived from titrated PC, spiked in neat serum at 1667–6.86 ng/mL and processed through the ACE and affinity assay workflow. The free ADA LBA signal window must be high enough to demonstrate a sufficient level of inhibition over the range of drug concentrations so *K*_*D*_ may be accurately calculated. Asterisks denote that the 6.86 ng/mL spiked PC affinity curve was the only PC concentration to fail the inhibition “flatness” test criteria; all other spike levels (1667–20.6 ng/mL) passed; (**B**) PC affinity curves with and without PF-06480605, 25 μg/mL

To confirm whether the Erenna free ADA LBA method had sufficient sensitivity to determine affinity in patient samples containing various amounts of ADA, the following experiment was conducted. MAb PC against PF-06480605 was spiked into human serum at a range of concentrations (1667–6.86 ng/mL) and processed through the ACE and affinity workflow. Resulting affinity curves are shown in Fig. 3A, which demonstrate that free ADA signal vs. PF-06480605 concentration relationships is dependent on the PC preparation. The sensitivity of the free ADA LBA for affinity determination was ascertained as the lowest-spiked PC level to generate an affinity curve of sufficient quality to pass the flatness test, which was 20.6 ng/mL. PF-06480605 levels up to 25 μg/mL were well-tolerated in the free ADA assay and *K*_*D(geo)*_ values were generally similar for the PC with and without 25 μg/mL of the biotherapeutic (8.75 and 3.72 pM, respectively; Fig. 3B). The precision of the MAb PC *K*_*D*_ result (Table I) was determined over four separate assay runs using two different PC preps that were processed through the ACE procedure on different days. The *K*_*D*_ values were reproducible, ranging between 1.2 and 2.9 pM, with an overall 40% CV for the limited data set. Surprisingly, the MAb PC had a biphasic response showing 2 different affinity populations using the Erenna assay. When assayed on the Gyrolab, the PC showed a monophasic profile, as one would expect for a monoclonal antibody. Through the sample pre-treatment workflow, the PC (and ADA samples) are exposed to acid on three separate occasions, which may cause denaturation to some of the antibodies which could result in a population of varying affinities. Indeed, when the MAb PC was tested on Octet for acid dissociation and subsequent rebinding over multiple acid cycles, binding was reduced by 15–20% after each acid step (data not shown). This could be detected on a sensitive platform such as the Erenna and might be missed on the Gyrolab.

**Table I:** Precision of Anti-PF-06480605 MAb PC *K*_*D*_ in the ADA Affinity Assay

Precision of MAb positive control *K*_*D*_			
Experiment	Day	PC ACE prep	*K*_*D*_ (pM)
1	1	1	1.4
2	2	1	1.2
3	3	2	2.9
3	3	2	2.3
Mean *K*_*D*_			2.0
SD			0.80
%CV			41

The precision was determined over four separate assay runs using two different PC preps that were processed through ACE procedure on two separate days

### Clinical ADA Affinity Sample Analysis

**Fig. 4:**
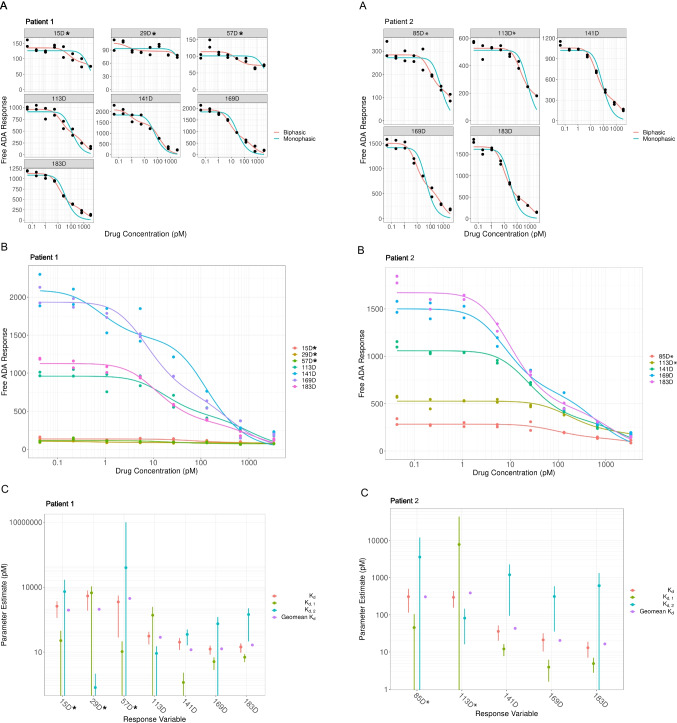
Clinical sample ADA *K*_*D*_ comparison throughout the study time course for two representative patients 1 and 2, respectively. Asterisks denote time points with less accurate *K*_*D*_ measurements, likely resulting in the profiles failing the inhibition “flatness” test criteria. (**A**) Fitted biphasic and monophasic affinity curves (time in days post treatment with PF-06480605) for each patient; (**B**) overlayed affinity curves (best fit) for each patient; and (**C**) *K*_*D*_ values: monophasic (*K*_*D*_), biphasic (*K*_*D,1*_ and *K*_*D,2*_), and geometric mean (Geomean *K*_*D*_) and confidence intervals throughout the study time course, per patient

A series of study incurred samples across the duration of the clinical trial was obtained from a subset of patients treated with PF-06480605 and assessed for ADA affinity. Samples were processed using the described ACE procedure. Briefly, a constant dilution of the enriched ADA sample was incubated with a range of PF-06480605 concentrations and incubated to equilibrium. Resulting solution phase equilibrium samples were analyzed using the Erenna ADA assay to determine free ADA response. Apparent *K*_*D*_ values were subsequently calculated by fitting affinity curves using a custom R Shiny application, and flatness test criteria were applied to assess the quality of the affinity profiles. Affinity curves were produced for multiple PF-06480605-treated patients with samples collected at several time points throughout the study. Two representative patient affinity profiles (patients 1 and 2) are shown in Fig. 4A and B. The overlaying line shows the best fit, monophasic or biphasic. Affinity data from all time points were included in the results, with notations for those affinity curves failing the flatness test acceptance criteria.

**Fig. 5:**
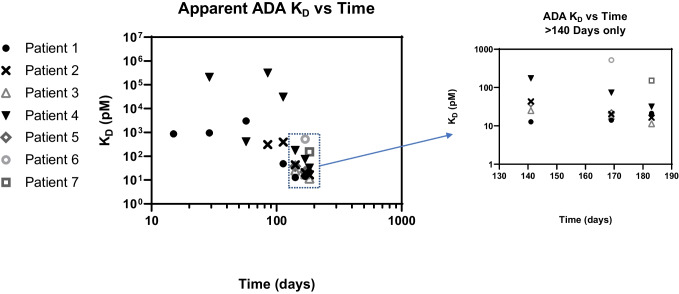
Apparent *K*_*D*_ measurements for 7 different patient samples analyzed showed increasing trend towards lower *K*_*D*_ (higher affinity) across the time course, indicating a maturing immune response to the PF-06480605 therapeutic candidate. Apparent affinity measurements increased from 20 to 10,000–fold depending on the patient subset tested from early to late time points. Inset graph (log-lin scale) included for late time points (> 140 days) to enhance data viewing

Figure 4C shows the calculated monophasic, biphasic, and geometric mean ADA *K*_*D*_ values for patients 1 and 2, with associated confidence intervals. The first three time points for patient 1 and first two time points for patient 2 (denoted with asterisks) had less accurate *K*_*D*_ measurements as indicated by larger confidence intervals, in addition to failing the inhibition curve “flatness” test. Figure 5 shows that for all patient subsets tested, the *K*_*D*_ measurements showed a trend towards lower *K*_*D*_ (higher affinity) as time progressed, consistent with a maturing immune response against PF-06480605. For early study time points, spanning 15 to 85 days, apparent *K*_*D*_ values ranged from 330 to 0.306 nM, whereas for later study time points, spanning 113 to 183 days, apparent *K*_*D*_ values ranged from 29.2 to 0.0114 nM. Overall, apparent affinity measurements increased from 20 to 10,000–fold, depending on the patient tested, from early to late time points in the clinical study. Additional data from a longer duration study would be needed to understand if further changes in *K*_*D*_ would be observed with long-term PF-06480605 treatment.

**Fig. 6:**
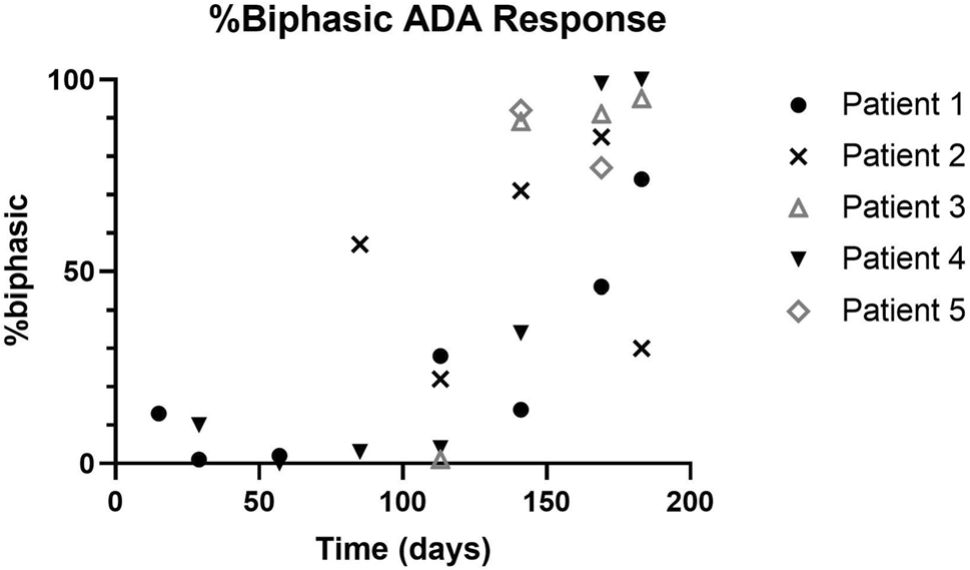
Nature of biphasic affinity response across the study time course for five patients with sufficient sampling times across the time course. For a large proportion of the patients tested (4/5), ADA responses were primarily monophasic (low % biphasic) in earlier time points trending to an increasingly biphasic best fit response over the time course, although the time switch from monophasic to biphasic varied for each individual

**Fig. 7:**
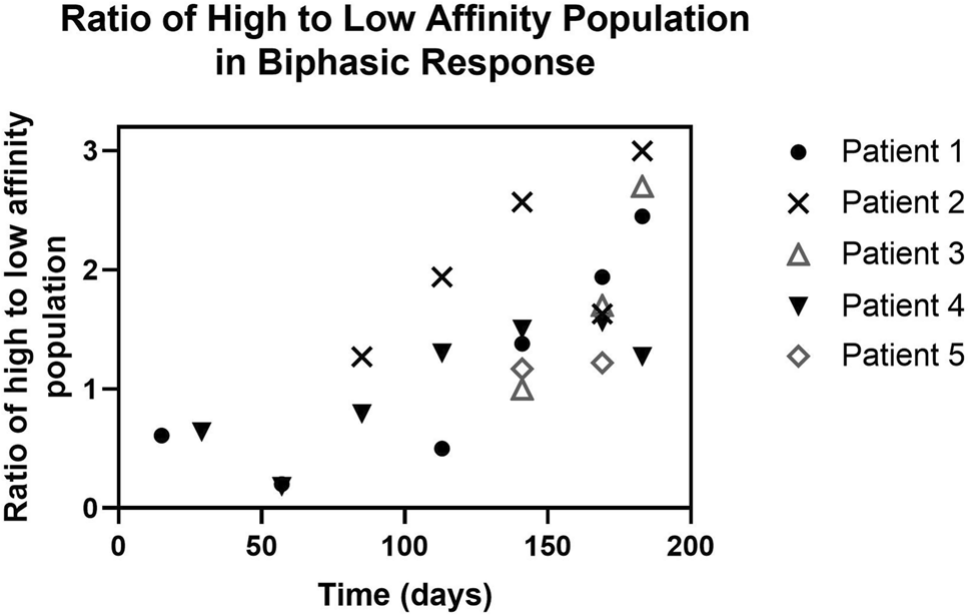
In the biphasic ADA responses for samples from patients 1–5, which had sufficient sampling time points, the proportion of high affinity ADA population compared to the low affinity ADA population increased over time in four out of the five patients, as depicted in Fig. 7

For a considerable proportion of patients with multiple time points (4/5), the anti-PF-06480605 antibody responses were primarily monophasic in the earlier time points, based on best-fit analysis, and trending to a biphasic dominant response later in the study (Fig. 6). The time switch from monophasic to biphasic response varied for each patient. Moreover, for the biphasic responses, the proportion of high-affinity ADA population compared to the low affinity ADA population increased over time in patients 1–4 and stayed approximately equal in proportion for patient 5 (only two time points tested), as depicted in Fig. 7.

**Fig. 8:**
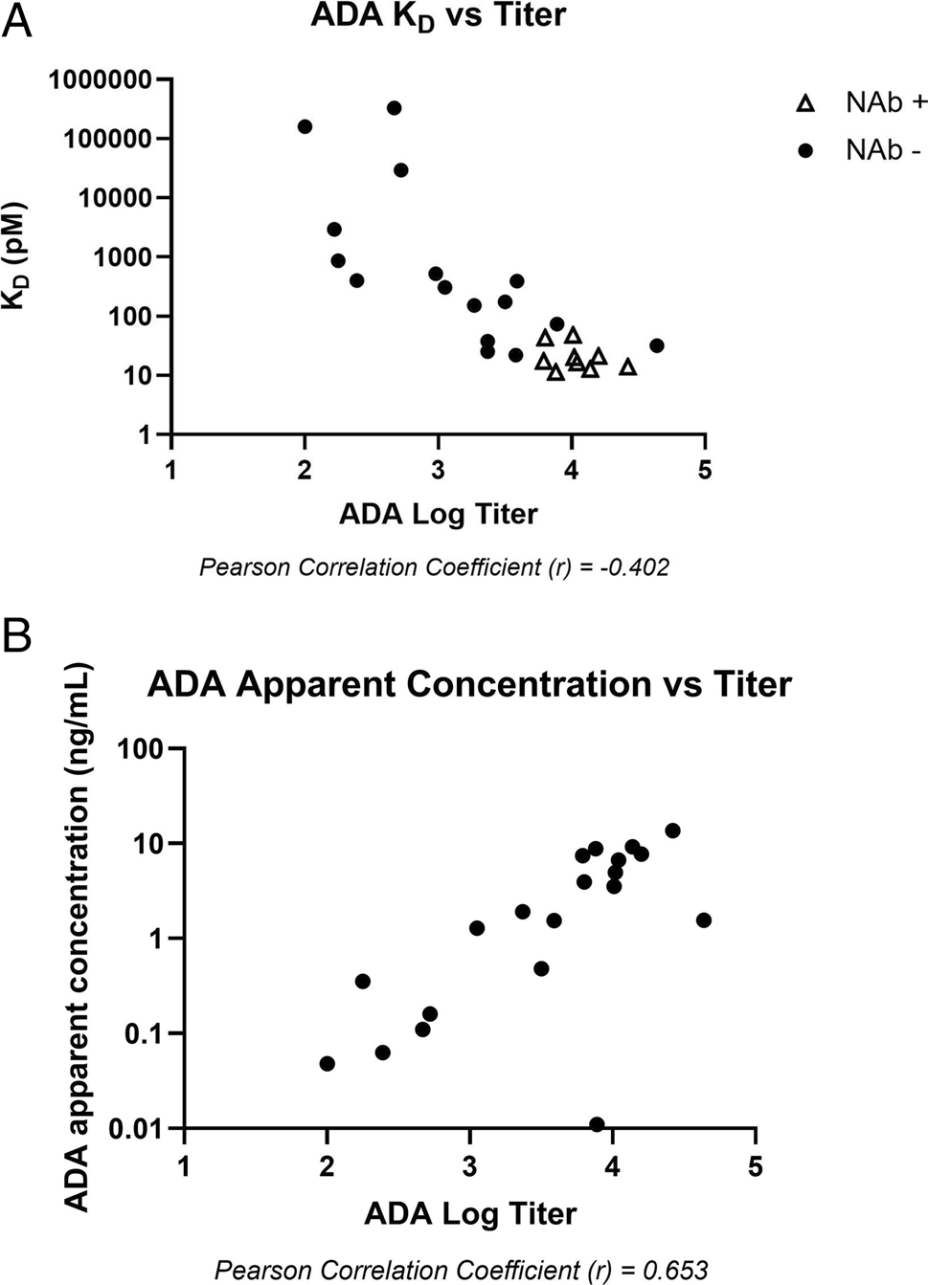
(**A**) Apparent *K*_*D*_ levels from all seven patients across the time course were plotted against the log titer ADA data. Samples that were NAb negative are closed circles; NAb positive samples are open triangles; (**B**) apparent ADA concentrations are plotted against ADA titer data from four patients

Apparent *K*_*D*_ values calculated for ADA samples were also plotted against ADA titers (Fig. 8A). Briefly, ADA to PF-06480605 were detected using a standard bridging LBA format on Mesoscale Discovery (MSD) platform. ADA formed complexes between biotinylated and ruthenium-labeled drug, which were then captured on streptavidin-coated MSD plates and detected by electrochemiluminescence. Figure 8A shows a clear trend of decreasing *K*_*D*_ (increasing affinity) as ADA log titer increased (Pearson correlation coefficient (*r*) =  − 0.402). Neutralizing antibody activity to PF-06480605 was also assessed in the study using a cell-based assay (22). Consistent with high titer and high affinity observed, neutralizing antibody activity was also evident (Fig. 8A). In addition, ADA log titer positively correlated with apparent ADA concentration, as shown on Fig. 8B (Pearson correlation coefficient (*r*) = 0.653).

## Discussion

Characterization of clinical anti-drug antibody (ADA) responses to biotherapeutics can be important to understanding the consequences of immunogenicity. ADA are expected to be polyclonal in nature, and the polyclonality of the response includes various epitope specificities, isotype classes, and a range of binding affinities to a given epitope. Here, we applied a novel solution-based equilibrium bioanalytical approach to determine an apparent ADA binding affinity (*K*_*D*_), further characterizing the clinical ADA response to PF-06480605, a monoclonal antibody biotherapeutic. Free ADA from solution affinity samples were detected using a sensitive LBA bridging format on the Singulex Erenna platform and characterized using a custom-designed interactive R Shiny application to calculate an apparent *K*_*D*_ value. The affinity model assumed a biclonal population (low and high affinity populations) and included additional outputs beyond *K*_*D*_, including (1) monophasic vs biphasic best-fit profiles; (2) a single geometric mean *K*_*D*_ output (*K*_*D(geo)*_) to account for both high and low affinity populations, and (3) the proportion (%) attributed to high vs low affinity antibody populations in the biphasic model. We applied a flatness test to determine the quality of the *K*_*D*_ output and used this as acceptance criteria for each affinity curve. For illustrative purposes, we included affinity data from all patient time points, denoting where affinity curves failed acceptance criteria. Affinity curves tended to fail the acceptance criteria for early time points only (≥ 113–140 days), where the ADA affinity and/or concentration was too low to be accurately measured by the free ADA LBA assay. Even though these data did not pass our criteria for data quality, the low LBA signal and flat curve signified low affinity and was worth including in the data set, with appropriate denotation on the figures as appropriate.

The resulting ADA assay demonstrated drug tolerance up to 25 μg/ml. The overall affinity determination method produced *K*_*D*_ values similar to those obtained using KinExA, a broadly accepted platform, when applied to the PC reagent against PF-06480605. The procedure was sufficiently sensitive to measure apparent ADA affinity in study incurred ADA-positive clinical samples. Samples collected from 7 patients at various time points during the study allowed for a longitudinal analysis of *K*_*D*_, which decreased over time, consistent with a maturing immune response. Additionally, *K*_*D*_ values inversely correlated with ADA titers demonstrating a clear trend in increasing affinity as ADA log titer increased. Notably, NAb activity appeared at the intersection of high affinity and high titer. Given that there was also a positive correlation between log ADA titers and ADA apparent concentration, these data support the notion that ADA titers determined in ADA LBA methods are dependent on both the concentration of ADA and the affinity of the polyclonal ADA population. It should be noted that there are caveats to measuring apparent ADA concentrations; for example, the PC is a monoclonal antibody vs. polyclonal nature of ADA found in study samples. The evidence of assay signal response parallelism between PC and ADA positive samples was not assessed in this study.

Our data show that for the majority of patients with sufficient sampling time points (4 of 5 patients), there was a notable shift from a monophasic to a biphasic best fit as the time course progressed, and the proportion of high affinity ADA increased later in the study, up to threefold depending on the patient, as compared to the earlier time points. We suspect that the monophasic appearance of the antibody response at early time points was due to the detection of the higher affinity component of the polyclonal population, whereas lower affinity antibodies in this timeframe were likely below the free ADA LBA method detection limit. As the apparent affinity increased over time, the free ADA assay could detect more high affinity antibodies, which allowed differentiation of high vs. low affinity components. This was manifested in a reported biphasic best fit at later time points and a marked overall decrease in the geometric mean affinity value over the study time course for all patients, indicative of a maturing immune response. The change in the geometric *K*_*D*_ varied between patients, ranging from an approximately 20 to 10,000–fold increase in affinity, resulting in apparent affinities in the pM range. For the 5 of 7 patients with greater than 2 sampling time points across the study, the apparent *K*_*D*_ reached 200 pM or less by approximately 5 months (day 141) and 50 pM or less by 5 to 6 months with most values in the 11 to 30 pM range.

The ADA affinity evaluation protocol was initially developed using the Gyrolab platform due to the small sample volume requirements and high assay throughput. The platform offers an advantage in optimizing the sample pre-treatment workflow, as shown on Fig. 1, where we aimed to maximize the recovery of PC reagent from sample matrix (serum) and to improve ADA assay drug tolerance. However, the Gyrolab platform was determined to be insufficiently sensitive to detect ADA in study-incurred clinical samples that were previously processed through the ACE workflow. This is potentially due to short assay contact times, which may cause the Gyrolab platform to favor high affinity over low affinity interactions and the inability of the platform to detect very low concentrations of ADA.

Various platforms have been used and reported to determine mass-unit based ADA concentrations in samples, including ELISA and SPR biosensor, where affinity-purified polyclonal and monoclonal antibody reagents have been applied as the assay reference materials (23–25). The reported ADA concentrations have varied between studies and methods and generally were in the low to mid µg/mL range. In our present approach, we recognize the caveats to using a MAb PC surrogate standard given that sample ADAs have a polyclonal composition and ADA recovery during sample pre-treatment steps may differ, depending on ADA affinity. However in comparison, apparent ADA concentrations from our ACE procedure-enriched clinical samples scored in the ng/mL range, as opposed to the µg/mL range as referenced above. It should also be noted that prior investigations lacked enrichment methods or acid treatment, and many of the apparent ADA concentrations using SPR or ELISA platforms were below the level of quantitation. We also demonstrated a positive correlation between log ADA titers and apparent ADA concentration, which is not surprising given higher concentration of ADA would likely have increased signal in the ADA LBA resulting in higher titer value. Apparent ADA concentrations were estimated using a MAb PC reagent as a reference, as discussed previously. It is understood that PC material has a particular *K*_*D*_ (2 pM) and ADA found in patient samples have a diverse array of affinities, making the concentration evaluation semi-quantitative. Sample pre-treatment procedures and assay conditions may skew recovery and detection of high vs low affinity antibodies. The low recovery of the MAb PC reagent post-ACE procedure (30%) highlights the challenges of working with very high-affinity antibodies which may be difficult to elute and may lead to under-estimating ADA affinity in samples. Conversely, wash steps during assay incubation times may lead to a loss of low affinity antibodies, thereby skewing to higher affinity recovery. For the method described herein, a rabbit polyclonal PC was also processed through the ACE procedure resulting in 75–80% recovery (data not shown), however lacked the necessary drug tolerance, and was abandoned in favor of the MAb PC. The rabbit polyclonal PC recovery may be more indicative of ADA in a patient sample, with different affinities and epitope specificities.

Although reporting of the ADA results in mass units may be suboptimal due to the suspected lack of parallelism to a MAb reference control and vast difference in the *K*_*D*_’s between PC and incurred samples found ADAs, it may still be a practical option. With all the caveats mentioned, there are benefits to reporting in mass units, such as direct comparison of ADA data between studies and compounds, particularly where we observe high titer and mature immune responses and when similar or the same PC reagent is utilized. Given the high sensitivity of the Erenna, another benefit is the ability to semi-quantitatively detect ADA antibody concentrations in the ng/mL range and enable reporting of ADA results in mass units if warranted or desired.

**Fig. 9:**
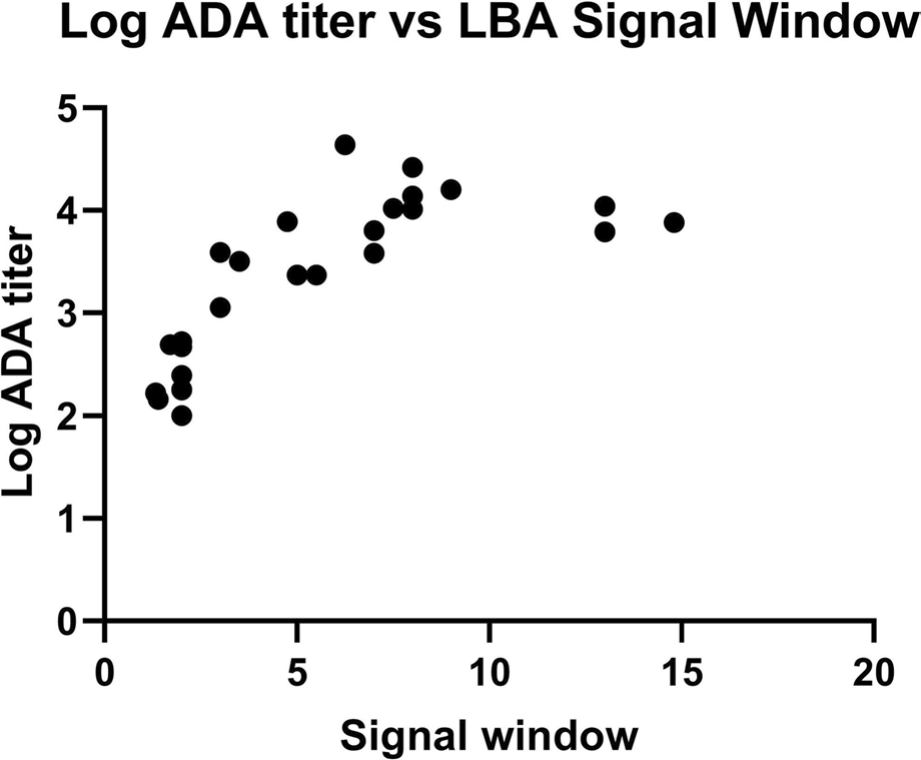
Relationship of log ADA titer and free ADA LBA signal window. The assay signal window is the ratio of the highest free ADA LBA signal (lowest drug level) and lowest free ADA LBA signal (highest drug level) for each sample solution affinity curve. In general, *K*_*D*_ measurements were more accurate and precise for samples with ADA log titers ≥ 3.0 and assay signal window ≥ 4.0

Although the Erenna was shown to be 125-fold more sensitive than the Gyrolab platform and could successfully detect apparent sample ADA at low concentrations and determine apparent ADA affinities, there were some notable limitations. Low-affinity ADA could be detected at the earlier time points; however, the low LBA assay signal observed resulted in imprecise *K*_*D*_ determinations, as demonstrated by an increase in confidence interval limits and failed inhibition curve (flatness) acceptance test. In general, *K*_*D*_ measurements were more accurate and precise for samples with ADA log titers ≥ 3.0 and assay signal window ≥ 4.0. The assay signal window is the ratio of the highest free ADA LBA signal (lowest drug level) and lowest free ADA LBA signal (highest drug level) for each sample solution affinity curve. ADA log titer and assay signal window parameters are positively correlated as shown on Fig. 9. Samples with ADA log titer values lower than 3.0 may contain low antibody concentrations, low affinity ADA constituents or both. However, even with the assay and platform limitations, the sensitivity afforded by the Erenna allowed us to successfully compare apparent affinities within and between a small set of patients across a time course, and various trends and correlations shown over the time course related to apparent affinity and other immunogenicity parameters (e.g., ADA titer, NAb activity) were very strong. The access to an even higher sensitivity analytical platform or further improving Erenna platform sensitivity may further advance our ability to determine *K*_*D*_ values at early time points with potentially low ADA concentrations and affinity values.

Although the high affinity of the MAb PC reagent (K_D_ ~ 2 pM) did not directly represent affinities of all ADAs detected in in this investigation (0.010–330 nM), the higher affinities measured at the later time points, ranging from 174 to 11 pM, certainly fall within the range of the MAb PC K_D_. To that end, mid-high affinity ADA (~ 1000–10 pM) detected in the study are relevant to the *K*_*D*_ of the PC used in the assay and PC affinities in this range would be appropriate.

## Conclusion

Characterization of apparent ADA affinity in patient samples can complement the existing repertoire of immunogenicity data, including titer, NAb activity, and isotype analysis, which may further inform the clinical immune response and its relevance to drug safety, PK, pharmacodynamic responses, and efficacy. In addition, these valuable “real-world” data can be useful comparators or inputs to novel modeling and simulation approaches designed for predicting clinical immunogenicity incidence and consequence during drug discovery and development. Despite several challenges, we demonstrated the ability to develop a workflow and LBA method with sufficient sensitivity to measure and compare apparent ADA affinity across a time course in a subset of clinical patient samples. The workflow, assay, and statistical tools could be utilized to further characterize immunogenicity against other biotherapeutics and thereby advance out understanding of the clinical consequences of unwanted ADA towards therapeutics.
